# Kiestein as an Evidence of Pregnancy

**Published:** 1842-04

**Authors:** H. Letheby


*Kiestein as an evidence of Pregnancy.* By H. Letheby, A. L. S.—In No. 11 of the Guy's Hospital Reports, there is a valuable paper upon this subject, by Dr. Bird, in which he enumerates many cases to prove the existence of this principle in the urine of pregnant women. Since the publication of that report I have had many opportunities of investigating the subject; and, as the result will show, it forms an important addition to the already known symptoms of pregnancy. The object of this paper, however, is not only in furtherance of its value as such a test, but to point out certain precautions to be observed in the experiments, in order to prevent fallacy.

The urine should be procured at a time when the woman is as free from disease as possible; and I believe that passed early in the morning, after rest, gives the least variable indications. This should be exposed, in a tall narrow glass to a continuous temperature of about 70° of F.; if a much lower temperature than this is used, say about 40°. I have known the urine stand for more than a fortnight without undergoing any change, although it be replete with kiestein or its principles, at a temperature of 70° However, if the woman be pregnant, we shall observe, in two or three days, the first indication of its presence, by the urine becoming turbid. In a day or two more a thin pellicle forms on its surface, and this gradually acquires consistence up to a fortnight from the onset of the experiment. But long before this time you will have noticed its characteristic odor; certainly not like cheese, to which Dr. Bird compares it, but precisely analogous to the smell of raw beef beginning to putrefy: it is emphatically a *putrid smell.* I have kept the urine more than a month after this, but it never loses either its pellicle or peculiar odor.

Besides the error likely to arise from the adoption of too low a temperature, where the kiestein would not be separated, I would warn the earlier experimenter not to fall into the opposite error of confounding the pellicle which forms upon all urine on standing, especially that which contains the lithates in excess; the more so as the general as well as microscopic appearance of this pellicle is often precisely like that of kiestein. The appearance I am now alluding to, however, is never accompanied with the putrid animal odor; but, on the contrary, gives out a copious smell of ammonia, and when disturbed falls immediately to the bottom of the liquid. These are the two especial distinctions.

On the value of this test I shall be very brief:—Of the 30 cases examined by Dr. Bird, 27 gave the required indications of the presence of kiestein; the other 3 were at the same time suffering under febrile excitement. Dr. Bird could not detect it in the urine of unimpregnated women, or after parturition, and during suckling

In the American Medical Library, as quoted by the British and Foreign Medical Review for October last, is a report of the experiments of Drs. M'Pherters and Perry, the resident physicians at Philadelphia Hospital. These gentlemen found it in the urine of 24 out of 27 pregnant women. Of the three negative cases, two were not in health when experimented on; further, they could not detect it in the urine of 27 unimpregnated women.

In my own experiments, which have been made at all dates between the second and ninth month of utero-gestation, there was unquestionable evidence of kiestein in 48 out of 50 cases. I am unable to account for its absence in the two exceptions, for I took care at all times to have the urine as free from disorder as possible.

In 17 non-pregnant women there was no indication of its presence. In examining the urine of 10 women during the time of suckling, I found it in all immediately after delivery, but that the evidence of its existence fell off at a period between the second and six months.

A question now naturally arises as to the cause of the presence of this principle, and what is its composition? It appears easily accounted for on the known sympathy that exists between the uterus and the breasts; the latter of which, taking cognizance of the gravid condition of the uterus, prepares itself betimes for the proper performance of that function which by and by is to become its necessary duty. Certain principles analogous to those of milk being imperfectly secreted, may, in this nascent condition, become reabsorbed, because, as Dr. Bird suggests, they do not find a ready outlet, and getting into the blood, are excreted thence by the kidneys; and this habit of reabsorption may go on for some little time after the birth of the child.

The composition of kiestein is not so easily made out: examined by the microscope it consists at first of a multitude of globules, varying in size from the one thirty-two thousandth to the one eight thousandth of an inch; after a time these break up, or coalesce and form flakes, and then crystals of triple phosphate generally become pretty abundant in it. This shows that the greasy appearance of the pellicle is not due, as Dr. Bird supposes, to the triple phosphate, for this is after formation; nor are these globules composed of fat, for they are perfectly insoluble in ether. I have not been able to detect them in the urine until it becomes turbid, so that they appear to be formed in the urine after expulsion. They are soluble in alkalies and in boiling acetic acid, and give all the reactions characteristic of coagulated albumen or fibrin: to these, then, they are most analogous; but nothing but an ultimate analysis can determine their identity or not. The globules do not differ in appearance from those contained in milk, but their complete insolubility in ether shows that they do differ.—*Am. Med. Intelligencer*, from *Lond. Med. Gaz.*

